# Human Secreted Ly-6/uPAR Related Protein-1 (SLURP-1) Is a Selective Allosteric Antagonist of α7 Nicotinic Acetylcholine Receptor

**DOI:** 10.1371/journal.pone.0149733

**Published:** 2016-02-23

**Authors:** Ekaterina N. Lyukmanova, Mikhail A. Shulepko, Denis Kudryavtsev, Maxim L. Bychkov, Dmitrii S. Kulbatskii, Igor E. Kasheverov, Maria V. Astapova, Alexey V. Feofanov, Morten S. Thomsen, Jens D. Mikkelsen, Zakhar O. Shenkarev, Victor I. Tsetlin, Dmitry A. Dolgikh, Mikhail P. Kirpichnikov

**Affiliations:** 1 Biological Department, Lomonosov Moscow State University, Moscow, Russian Federation; 2 Department of Bioengineering, Shemyakin and Ovchinnikov Institute of Bioorganic Chemistry, Russian Academy of Sciences, Moscow, Russian Federation; 3 Department of Molecular Basics of Neurosignalling, Shemyakin and Ovchinnikov Institute of Bioorganic Chemistry, Russian Academy of Sciences, Moscow, Russian Federation; 4 Department of Structural Biology, Shemyakin and Ovchinnikov Institute of Bioorganic Chemistry, Russian Academy of Sciences, Moscow, Russian Federation; 5 Department of Drug Design and Pharmacology, University of Copenhagen, Copenhagen, Denmark; 6 Neurobiology Research Unit, University Hospital, Copenhagen, Copenhagen, Denmark; 7 Moscow Institute of Physics and Technology (State University), Dolgoprudny, Moscow Region, Russian Federation; University of São Paulo, BRAZIL

## Abstract

SLURP-1 is a secreted toxin-like Ly-6/uPAR protein found in epithelium, sensory neurons and immune cells. Point mutations in the *slurp-1* gene cause the autosomal inflammation skin disease Mal de Meleda. SLURP-1 is considered an autocrine/paracrine hormone that regulates growth and differentiation of keratinocytes and controls inflammation and malignant cell transformation. The majority of previous studies of SLURP-1 have been made using fusion constructs containing, in addition to the native protein, extra polypeptide sequences. Here we describe the activity and pharmacological profile of a recombinant analogue of human SLURP-1 (rSLURP-1) differing from the native protein only by one additional *N*-terminal Met residue. rSLURP-1 significantly inhibited proliferation (up to ~ 40%, EC_50_ ~ 4 nM) of human oral keratinocytes (Het-1A cells). Application of mecamylamine and atropine,—non-selective inhibitors of nicotinic acetylcholine receptors (nAChRs) and muscarinic acetylcholine receptors, respectively, and anti-α7-nAChRs antibodies revealed α7 type nAChRs as an rSLURP-1 target in keratinocytes. Using affinity purification from human cortical extracts, we confirmed that rSLURP-1 binds selectively to the α7-nAChRs. Exposure of *Xenopus oocytes* expressing α7-nAChRs to rSLURP-1 caused a significant non-competitive inhibition of the response to acetylcholine (up to ~ 70%, IC_50_ ~ 1 μM). It was shown that rSLURP-1 binds to α7-nAChRs overexpressed in GH_4_C_l_ cells, but does not compete with ^125^I-α-bungarotoxin for binding to the receptor. These findings imply an allosteric antagonist-like mode of SLURP-1 interaction with α7-nAChRs outside the classical ligand-binding site. Contrary to rSLURP-1, other inhibitors of α7-nAChRs (mecamylamine, α-bungarotoxin and Lynx1) did not suppress the proliferation of keratinocytes. Moreover, the co-application of α-bungarotoxin with rSLURP-1 did not influence antiproliferative activity of the latter. This supports the hypothesis that the antiproliferative activity of SLURP-1 is related to ‘metabotropic’ signaling pathway through α7-nAChR, that activates intracellular signaling cascades without opening the receptor channel.

## Introduction

A number of endogenous ligands acting on nicotinic acetylcholine receptors (nAChRs) and belonging to the Ly-6/uPAR family were discovered in higher animals [1]. These proteins share structural homology with ‘three-finger’ snake α-neurotoxins, specific inhibitors of nAChRs [1,2]. Some of these endogenous ligands (Lynx1, Lynx2, Lypd6) are membrane-tethered via GPI-anchor and co-localize with nAChRs, thus modulating their functions in the brain [3–6], while others like Secreted Ly-6/uPAR Related Protein-1 and -2 (SLURP-1 and SLURP-2) are secreted proteins [7,8].

Human SLURP-1 was initially isolated from blood and urine libraries [7]. Point mutations in the *slurp-1* gene cause the autosomal inflammation skin disease Mal de Meleda [9]. Using recombinant analogue of SLURP-1 it was proposed that SLURP-1 acts as allosteric modulator and potentiates ion currents through α7-nAChRs in the presence of acetylcholine (ACh) [10]. SLURP-1 participates in the regulation of keratinocyte proliferation and differentiation, supposedly via interaction with α7-nAChRs, and may function as an autocrine/paracrine hormone in epithelium [11,12]. It was shown that SLURP-1 activates protein kinase signaling cascade resulting in up-regulation of nuclear factor-κB expression in keratinocytes [13]. Expression of SLURP-1 in immune cells and its anti-inflammatory effects on human intestinal epithelial cells and immunocytes have been described [14–16]. Moreover, SLURP-1 is expressed in sensory neurons and might be involved in the cholinergic pain modulation within the spinal cord [17]. Recently, SLURP-1 expression was detected in HT-29 human colorectal adenocarcinoma cells, and the SLURP-1 expression level in these cells was significantly suppressed upon nicotine treatment [18]. Application of a recombinant SLURP-1 analogue to these cells resulted in a significant reduction of cancer cell proliferation [19].

**Fig 1:**
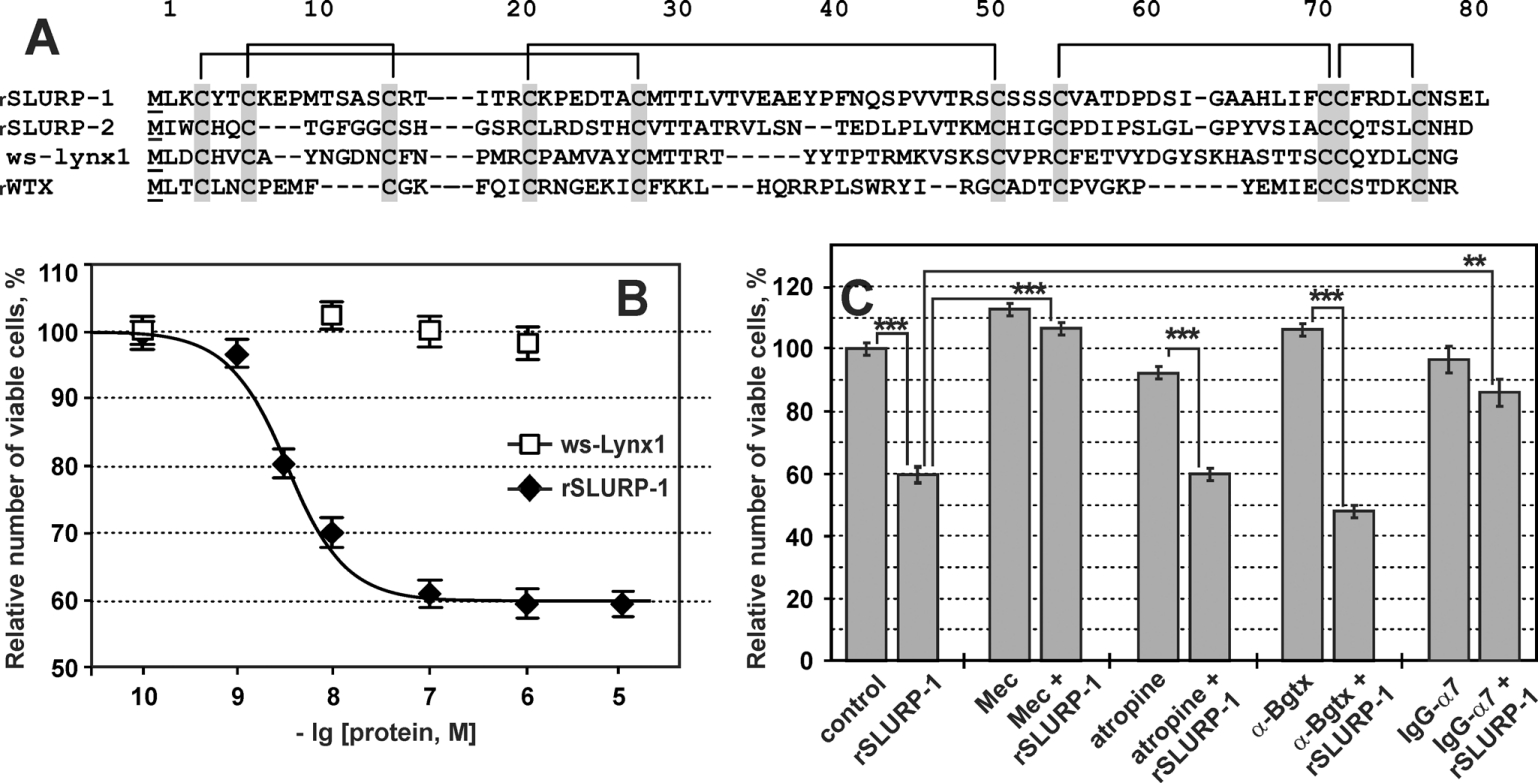
Effect of rSLURP-1 on the growth of Het-1A cells. (A). Amino acid sequence alignment of human SLURP-1, SLURP-2, ws-Lynx1, and non-conventional toxin WTX from *N*. *kaouthia*. Cysteine residues are labeled in gray, and the disulfide linkages are shown; additional *N*-terminal Met residues are underlined. (B). Influence of rSLURP-1 (diamonds) and ws-Lynx1 (squares) on cell growth. Each point is mean ± S.E. of six independent experiments. The Hill equation (Eq 1) was fitted to rSLURP-1 data (% of control) for each of the six experiments independently. After averaging the following values for EC_50_, nH and A1 were obtained 4.3 ± 0.6 nM, 1.4 ± 0.2 and 59.5 ± 1.3% (mean ± S.E., n = 6). (C). Effects of rSLURP-1 (1 μM), atropine (1 μM), Mec (10 μM), α-Bgtx (1 μM), polyclonal antibodies against α7 (IgG-α7, 1 μg per 50 μl), and their co-application. Each bar is mean ± S.E. of 4–6 independent experiments. ** and *** indicate significant (p<0.01 and p<0.001, t-test) differences.

In spite of the growing evidences supporting a modulatory action of SLURP-1 on nAChR function, the current knowledge about the mechanism of SLURP-1/nAChR interactions is very limited. The progress in this field is hampered by the inability to extract sufficient amounts of SLURP-1 from natural sources and difficulties in the production of the recombinant protein with native sequence and structure. The majority of previous works on SLURP-1 were done using fusion constructs containing, in addition to SLURP-1, some polypeptide fragments, e.g. *N*-terminal SUMO protein, MPB protein, GST protein or *C*-terminal Myc-tag [10–13,16,20–22]. It was shown previously that the native SLURP-1 isolated from blood (MW 8,842 Da, 81 a.a.) and a recombinant SLURP-1 analogue produced in HEK-293 cells (yield ~ 0.1 mg/l of cell culture) were not glycosylated [7,10]. This fact points to the possibility to use bacterial expression systems for recombinant production of SLURP-1. Previously we developed the high-efficient *E*. *coli* expression system for protein analogue with the near-native structure (rSLURP-1, MW 8,974 Da, 82 a.a.) [23]. The only difference of rSLURP-1 from the native protein is the additional *N*-terminal Met residue appearing due the cloning of *rslurp-1* gene into the expression vector, Fig 1A. The relatively high yield of the recombinant production (~ 5 mg of the refolded protein from 1 l of cell culture) allowed us to carry out NMR structural study of rSLURP-1, which ultimately confirmed its structural homology with the ‘three-finger’ snake neurotoxins and Lynx1, another ‘three-finger’ human neuromodulator acting on nAChRs (Fig 1A) [23].

Here we describe functional properties of rSLURP-1. Our data provide a new insight into the mechanism of SLURP-1 action and reveal some discrepancies with the results of previous studies obtained using SLURP-1 analogues with non-native sequences. For example, our findings demonstrate that SLURP-1 acts on α7-nAChRs as an allosteric antagonist and interacts with the receptor outside the classical ligand-binding site.

## Materials and Methods

Water-soluble domain of human Lynx1 (ws-Lynx1), rSLURP-1, and rSLURP-2 were produced in *E*. *coli* as described in [19, 23, 24]. The purity and homogeneity of the recombinant proteins were confirmed by SDS-PAGE, HPLC, and MALDI-MS. The disulfide bonds formation was confirmed in the reaction with the Ellman’s reagent (5,5′-dithio-bis(2-nitrobenzoic acid). The correct spatial structure for each batch of produced proteins was confirmed by NMR-spectroscopy.

### Experiments with Het-1A cells

Human Het-1A squamous esophageal cells were obtained from American Type Culture Collection (ATCC, CRL-2692). Cells were cultured at 37°C and 5% CO_2_ in BEBM media (Lonza/Clonetics Corporation) according to ATCC recommendations. Culture flasks and plates were precoated with a mixture of 0.01 mg/mL fibronectin, 0.03 mg/mL bovine collagen type I and 0.01 mg/mL bovine serum albumin dissolved in culture medium. Cells were seeded in 96-well plates (1×10^4^ cells per well), and 24 h later the analyzed compounds were added to cells. Next, cells were incubated for 48 h, examined under microscope, and characterized using WST-1 assay and fluorescent Hoechst/propidium iodide assay as described elsewhere [19,25]. Hoechst/propidium iodide assay allows evaluation of cell viability by staining all cell nuclei with Hoechst 33342 and dead cell nuclei with propidium iodide. For inhibition of nAChRs and muscarinic acetylcholine receptors (mAChRs), 1 h preincubation of keratinocytes with 1 μM α-bungarotoxin (α-Bgtx, Tocris), 10 μM mecamylamine hydrochloride (Mec, Sigma), 1 μM atropine (Sigma), or 1 μg per 50 μl anti-α7-nAChR antibody (#ab10096, Abcam, Cambridge, UK) was performed before rSLURP-1 application. Antibodies at the same concentration were additionally added to the cells after 24 and 40 hours of incubation with rSLURP-1.

### Affinity purification

Human temporal neocortical tissue was obtained from an anterior temporal lobectomy performed in two patients (females, age 30, and 44) with medically intractable temporal lobe epilepsy with hippocampal onset. Written informed consent was obtained before surgery. The study was approved by the Ethical Committee in the Capital Region of Denmark (H-2-2011-104) and performed in accordance with the Declaration of Helsinki. The tissue was immediately frozen on dry ice and stored at -80°C until use. The neuropathological examinations of the neocortex revealed no abnormalities.

rSLURP-1 was dissolved to 2 mg/ml in distilled H_2_O at 4°C for 48 hours after which it was coupled to PureProteome NHS Flexibind magnetic beads (Millipore, Billerica, MA) in a ratio of 1:2 (vol/vol) using the manufacturer’s instructions. Successful coupling was confirmed by subsequent protein determination showing a substantial decrease in the protein content of the applied rSLURP-1 solution. Another batch of beads was processed in parallel, but using PBS devoid of rSLURP-1, as a negative control. The beads were incubated in PBS supplemented with 0.1% bovine serum (pH 7.4, 1 hour at 4°C) prior to use.

The brain tissue was lysed in 1 ml lysis buffer (50 mM Tris, 50 mM NaCl, 5 mM EDTA, 5 mM EGTA, 10 μl/ml protease inhibitor cocktail (Sigma, pH 7.5) using a PT1200C polytron blender (Kinematica, Luzern, Switzerland) for 20 seconds. The lysate was centrifuged 30 minutes at 160,000×g at 20–22°C using an airdriven ultracentrifuge (Airfuge, Copenhagen, Denmark), and the supernatant discarded. The pellet was resuspended in 1 ml lysis buffer containing 2% Triton X-100 by blending for 20 seconds, and incubated 2 hours at 4°C on a rotor (15 rpm). Thereafter, the sample was centrifuged as above and the resulting supernatant was used for affinity purification. Total protein content was determined using the Pierce 660 nm Protein Assay (Thermo Scientific, Rockford, IL), and the protein (0.7–1.0 mg) was incubated with 50 μl magnetic beads in a total volume of 1.5 ml lysis buffer for 18–22 h at 4°C on a vertical rotor (15 rpm). The magnetic beads were separated using PureProteome Magnetic Stand (Millipore, Billerica, MA). Subsequently, the beads were washed twice in 1 M NaCl, 8 mM Na_2_HPO_4_, 2 mM NaH_2_PO_4_, 0.5% Triton X-100, pH 7.5 and three times in 0.1 M NaCl, 8 mM Na_2_HPO_4_, 2 mM NaH_2_PO_4_, 0.5% Triton X-100, pH 7.5 and immediately processed for Western blotting.

### Western blotting

Total protein content was measured using a DC Protein Assay Kit (Biorad, Hercules, CA). Amounts of lysates containing equal protein content were then diluted in loading buffer (120 mM Tris, 20% (v/v) glycerol, 10% (v/v) mercaptoethanol, 4% (w/v) SDS, 0.05% (w/v) bromophenol blue, pH 6.8), incubated for 10 minutes at 95°C, submitted to gel electrophoresis in AnykD gel (Biorad), and blotted onto polyvinylidene fluoride membranes (BioRad). Membranes were washed in Tris-buffered saline with 0.1% Tween 20 (TBST) and blocked in TBS containing 5% (w/v) dry milk powder, which was also used for antibody incubations. Incubation with primary antisera directed against β2 (1:1,000, provided by Dr. Cecilia Gotti), α3, α4, α5, α6, 5-HT_3_ (1:100 #sc-1771, sc-5591, sc-28795, sc-27292, sc-28958 Santa Cruz Biotechnology), α7, β4 (1:1,000 #ab23832 and 1:100 #ab156213 Abcam, Cambridge, UK) was performed overnight at 4°C on parafilm in a humidified container, followed by 3×10 minute washes in TBST and 1 h incubation at 21°C with horseradish peroxidase-conjugated secondary antibody (1:2,000, Dako, Glostrup, Denmark). After thorough washing in TBST, enhanced chemiluminescence Western blotting detection reagents (Western Lightning ECL Pro, Perkin Elmer, Waltham, MA) were used for signal detection and protein bands were visualized using a Chemidoc XR digital image analyser (Biorad).

### Electrophysiology

Wild-type mature female *Xenopus laevis* toads were purchased from Xenopus Express (France) and were treated according to guidelines of local bioethics committee. Each *Xenopus laevis* oocyte was injected with 2 ng of human α7 nAChR cDNA. Two-electrode voltage-clamp recordings were performed after 48–72 h of incubation at 18°C, followed by 1–7 days incubation at 4°C. Membrane potential was clamped at -60 mV, all recordings were performed using a hand-made chamber of 25 μL volume provided by Dr. C. Methfessel (Ruhr University, Bochum, Germany). Short 10 second pulses of ACh were applied to a receptor expressing oocyte each 5 minutes followed by washing out with control buffer after each pulse. To determine rSLURP-1 functional activity, we either performed 3 minute preincubation with rSLURP-1 or co-applied rSLURP-1 with ACh.

#### Analysis of binding at α7-nAChRs through competition with ^125^I-α-Bgtx

The tested amounts of rSLURP-1 (from 1 nM to 100 μM) were preincubated 3 h with the GH_4_C_l_ cells transfected with human α7 type nAChR (a gift of Eli Lilly Company) or with the *Lymnaea stagnalis* acetylcholine binding protein (Ls-AChBP, a gift from Prof.T. Sixma); final concentration of toxin-binding sites was 0.4 or 2.4 nM, respectively. Binding with the GH_4_C_1_ cells was carried out in 50 μl of 20 mM Tris/HCl buffer containing 1 mg/ml of BSA, pH 8.0, and with the Ls-AChBP—in 50 μl of binding buffer (PBS, containing 0.7 mg/ml of BSA and 0.05% Tween 20, pH 7.5) at 25°C. Next, ^125^I-α-Bgtx (~ 2000 Ci/mmol) was added to a final concentration of 0.2 nM for 5 min followed by filtration of a reaction mixture on GF/C filters (Whatman, Maidstone) presoaked in 0.25% polyethylenimine (for cells) or on double DE-81 filters (Whatman) presoaked in binding buffer (for Ls-AChBP). Unbound radioactivity was removed from the filters by washes (3×3 ml) with the respective incubation buffer. Nonspecific binding was determined in the presence of 10 μM α-cobratoxin (3 h preincubation).

### Analysis of the dose-response curves

The influence of rSLURP-1 on viability of Het-1A cells, on ion currents through α7-nAChRs expressed in *Xenopus* oocytes, and on the binding of ^125^I-α-Bgtx to Ls-AChBP was fitted by the Hill equation:

$$y=A1+\frac{100\%-A1}{1+{\left([rSLURP1]/IC_{50}\right)}^{nH}}$$ (eq. 1),

where [*rSLURP1*] is the rSLURP-1 concentration, IC_50_ is concentration where half-maximal inhibitory effect is achieved, nH is Hill coefficient, and the data (y) and the amplitude of the effect (*A1*) are expressed in the % of control.

## Results

### rSLURP-1 down-regulates keratinocyte proliferation

Incubation of Het-1A cells (immortalized line of human oral keratinocytes) with 1 μM rSLURP-1 for 48 h resulted in a significant decrease in the cell number up to 60 ± 2% relative to the control (Fig 1B). To discriminate between cytotoxicity and reduced proliferation, we performed additional microscopic examination of cells in plate wells and measured their viability with the Hoechst/propidium iodide assay. A decrease in the cell density was clearly observed, whereas morphology of most cells was not disturbed as compared to control (data not shown). Hoechst/propidium iodide assay did not reveal an increase in the fraction of dead cells even at the highest tested concentrations of rSLURP-1 (10 μM): 4 ± 1% of dead cells for both rSLURP-1 treated cells and in control. Therefore, rSLURP-1 affects Het-1A cells by reducing their proliferation. Analysis of the dose-response curve revealed a concentration-dependent mode of action for rSLURP-1 with an EC_50_ of 4.3 ± 0.6 nM (Fig 1B). For comparison, we analyzed cell growth in the presence of ws-Lynx1, another endogenous Ly-6/uPAR protein acting on nAChRs [26,27], at concentrations up to 1 μM (Fig 1B). No reduction in the number of cells was observed with ws-Lynx1.

To distinguish the effects of rSLURP-1 caused by possible interactions with nAChRs and mAChRs (both types of receptors are presented in keratinocytes [28]), we measured the antiproliferative activity of rSLURP-1 in the presence of a non-specific blocker of the nAChRs, Mec, and a non-specific antagonist of mAChRs, atropine (Fig 1C). We found that Mec did not influence cell growth per se, but completely inhibits the antiproliferative effect of rSLURP-1. Contrarily, atropine neither affected cell growth per se, nor inhibited the effect of SLURP-1. Incubation of keratinocytes with α-Bgtx (a competitive antagonist of muscle type, α7 and α9 nAChRs) also did not affect the cell growth and antiproliferative effects of rSLURP-1 (Fig 1C). To further investigate role of α7 nAChRs in the antiproliferative action of rSLURP-1 we measured the protein activity in the presence of anti-α7-nAChRs antibodies. It was found that application of anti-α7 antibodies did not influence cell growth, but significantly inhibits the antiproliferative activity of rSLURP-1 (the relative number of viable cells was increased from ~60% to ~85%, see Fig 1C).

### rSLURP-1 selectively binds to the α7 nAChR subtype in the human cortical extracts

**Fig 2:**
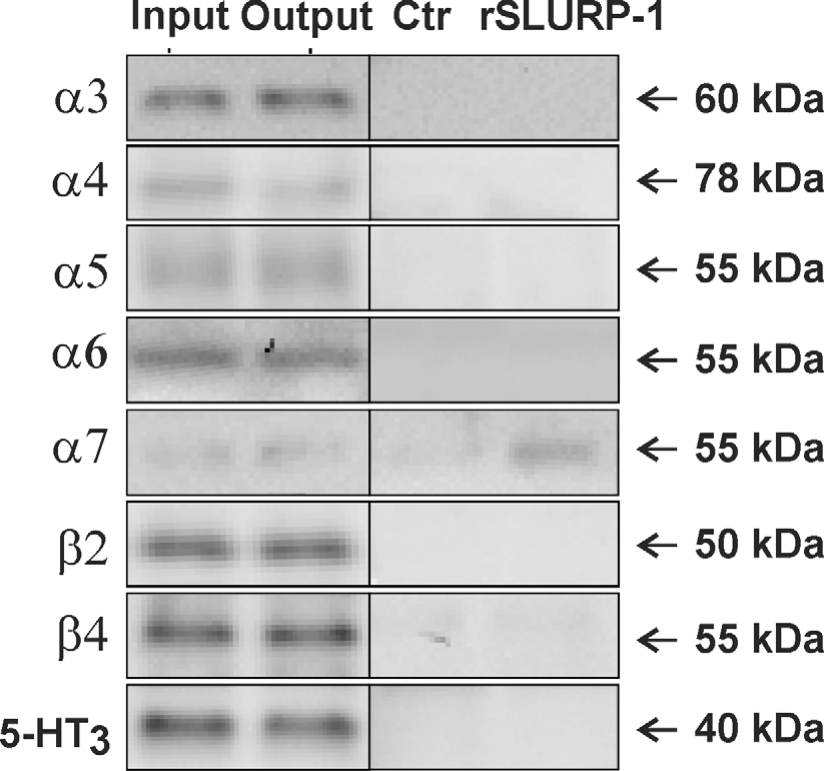
rSLURP-1 binds α7 nAChR subunits in the human brain. Affinity purification was performed using magnetic beads covalently coupled with rSLURP-1 or non-coupled beads (Ctrl) on human temporal cortical homogenates (n = 2). Samples were submitted to gel electrophoresis and Western blotting along with samples of the homogenate used for affinity purification (Input) and the remaining homogenate after affinity purification (Output) followed by detection of nAChR subunits.

According to previous measurements cultured keratinocytes contain relatively low number of nicotinic receptors [28]. Therefore to study the pharmacological profile of rSLURP-1 we chose other model system with larger receptor density, namely the extracts from temporal cortex. We performed affinity purification of different nAChR subunits from the extracts of the human temporal cortex using rSLURP-1 coupled to magnetic beads, followed by detection of nAChR subunits. rSLURP-1 isolated α7 nAChR subunits, but did not isolate the α3, α4, α5, α6, β2, or β4 subunits (Fig 2). Moreover bead-coupled rSLURP-1 did not isolate subunits of the closely related 5-HT_3_ receptor. None of the subunits were detected when the affinity purification was performed using non-coupled beads, confirming that isolation of α7 nAChR subunits was not attributable to a non-specific interaction with the beads.

### rSLURP-1 non-competitively inhibits ACh-evoked currents in α7 type nAChRs

**Fig 3:**
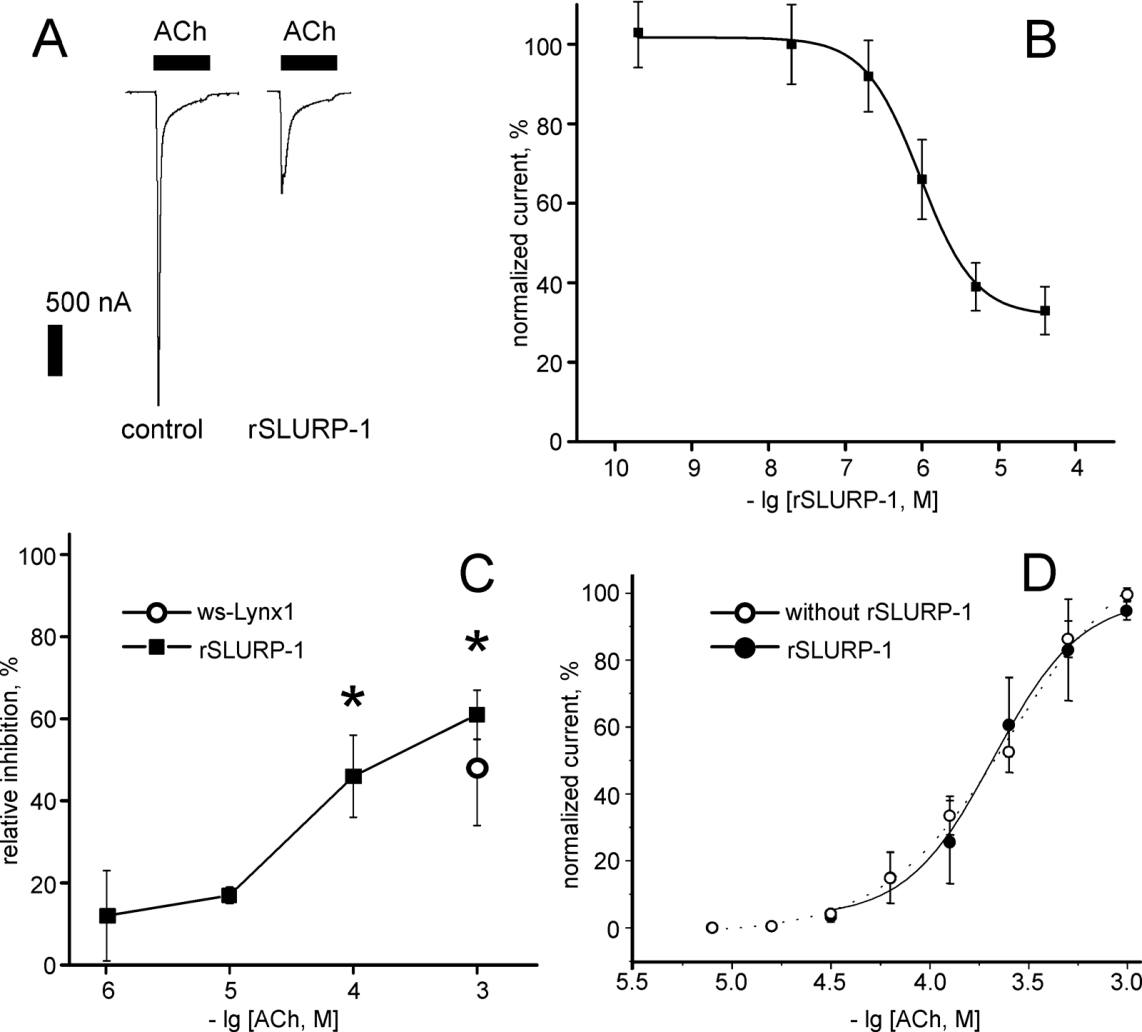
Inhibition of ACh-evoked current at α7-nAChR expressed in *X*. *laevis* oocytes by rSLURP-1. (A). Electrophysiological recordings of α7 nAChR inhibition by 13 μM rSLURP-1. Currents were obtained in response to 20 seconds 100 μM ACh pulses (horizontal bars). Inhibition of current amplitude was observed after 5 min pre-incubation with 13 μM rSLURP-1. Vertical bar represents current scale (500 nA). (B). Dose-response curve of 1 mM acetylcholine-evoked current by rSLURP-1. Each point is mean ± S.E. of independent measurements on three oocytes. The Hill equation (Eq 1) was fitted to normalized data (% of control) for each of the three experiments independently. After averaging the following values for IC_50_, nH and A1 were obtained 1.1 ± 0.5 μM, 1.4 ± 0.3 and 31 ± 3% (mean ± S.E., n = 3). (C). Dependence of 5 μM rSLURP-1 effect at α7-nAChR on ACh concentration. Data of rSLURP-1 induced inhibition is shown by filled squares, 5 μM ws-Lynx1 effect on 1 mM ACh is shown by circle. Asterisks indicate significant (p<0.05, t-test) difference from receptor inhibition by rSLURP-1 at 1 μM ACh (6 on plot). (D). Dose-response curves of acetylcholine-evoked current amplitudes in the absence (dotted line, empty circles) and presence (solid line, filled circles) of 1 μM rSLURP-1. The calculated parameters EC_50_ and nH were 232 ± 25 μM and 1.9 ± 0.3 in absence of rSLURP-1, and 214 ± 60 μM and 2.2 ± 0.3 in presence of rSLURP-1 (mean ± S.E., n = 3).

To characterize rSLURP-1 action, electrophysiological recordings were performed on *Xenopus* oocytes expressing human α7 type nAChRs in the absence and presence of 200 pM– 40 μM rSLURP-1 (Fig 3). At micromolar concentrations rSLURP-1 reduced ACh responses up to ~ 30% of control (IC_50_ = 1.1 ± 0.5 μM, Fig 3A and 3B). The inhibitory activity of 5 μM rSLURP-1 was tested using different ACh concentrations (1 μM– 1 mM, Fig 3C). Application of rSLURP-1 did not evoke currents in the absence of ACh. There was no detectable current amplitude reduction at low ACh concentrations, but it became significant (p<0.05, t-test) starting from 100 μM of ACh. This activity resembles the previously described inhibitory activity of ws-Lynx1 which effect also was more pronounced in the presence of high ACh concentration [26]. The direct comparison of the influence of 5 μM ws-Lynx1 and rSLURP-1 on the currents evoked by 1 mM ACh revealed similar level of inhibition (~ 50%, Fig 3C). Moreover, application of rSLURP-1 (1 μM) did not shift ACh dose-response curve revealing absence of competition between rSLURP-1 and ACh (Fig 3D). This strongly suggests that rSLURP-1 reduces ACh-evoked current amplitude in a manner consistent with an allosteric mode of action.

### rSLURP-1 does not compete with ^125^I-α-Bgtx for binding to human α7 type nAChRs

**Fig 4:**
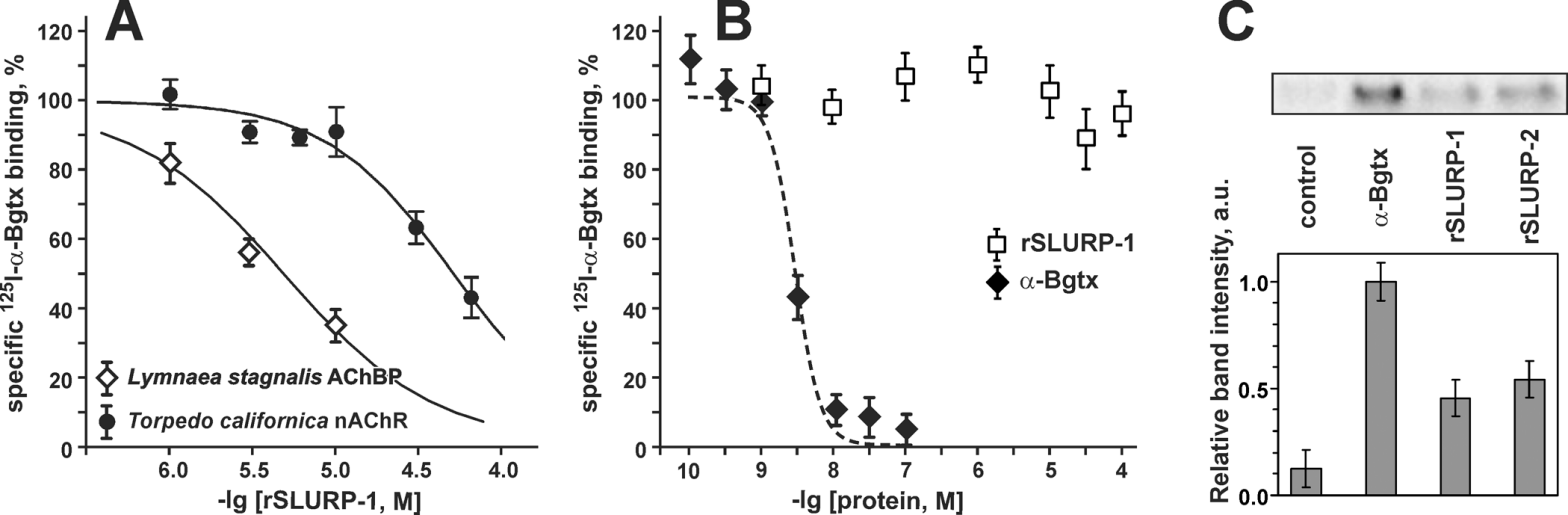
Competition of rSLURP-1 with ^125^I-α-Bgtx for binding to Ls-AChBP, membrane-bound nAChR from *Torpedo californica*, and α7-nAChR in the GH_4_C_1_ cell line. (A). Each point is mean ± S.E. of three independent experiments. The Hill equation (Eq 1, with A1 = 0%) was fitted to normalized Ls-AChBP and *T*. *californica* data (% of control binding) for each of the three experiments independently. After averaging the following values for IC_50_ and nH were obtained 4.8 ± 0.9 μM and 0.92 ± 0.17 for Ls-AChBP, and 54 ± 15 μM and 1.3 ± 0.3 for *T*. *californica* nAChR (mean ± S.E., n = 3). Data for *T*. *californica* were taken from [23]. (B). Displacement of ^125^I-α-Bgtx by unlabeled Bgtx and rSLURP-1 from α7-nAChR in the GH_4_C_1_ cells. (C). Affinity purification of α7-nAChR subunit was performed using magnetic beads covalently coupled with rSLURP-1, rSLURP-2, and α-Bgtx or non-coupled beads (control) on GH_4_C_1_ cells overexpressing α7-nAChR (n = 2). The 40 μl samples of GH_4_C_1_ cells with final concentration of α-Bgtx-binding sites of 0.4 nM were used. The blots were analyzed by densitometry using ImageJ software (http://imagej.nih.gov/ij/).

Previously, we demonstrated rSLURP-1 competition with ^125^I-α-Bgtx for binding to the muscle type nAChR from *Torpedo californica* with IC_50_ of 54 ± 15 μM (Fig 4A) [23]. Here the competition experiments with ^125^I-α-Bgtx yielded an IC_50_ of 4.8±0.9 μM for rSLURP-1 binding to Ls-AChBP, a structural homolog of the ligand-binding domain of nAChRs (Fig 4A). However, in case of human α7-nAChRs overexpressed in the GH_4_C_1_ cell line, no competition with ^125^I-α-Bgtx was detected for rSLURP-1 at concentration from 1 nM to 100 μM (Fig 4B). For comparison, the displacement of radioactive ^125^I-α-Bgtx by non-labeled α-Bgtx from α7-nAChRs in the GH_4_C_1_ cells is shown in the Fig 4B.

To confirm the binding of rSLURP-1 to α7-nAChRs overexpressed in the GH_4_C_1_ cells, we performed affinity purification of α7-nAChR subunits using rSLURP-1 coupled to magnetic beads. For comparison the magnetic beads with attached α-Bgtx and rSLURP-2 were also used. It was found that all three proteins are able to extract α7 subunit from GH_4_C_1_ cells with relative activity in the order α-Bgtx > rSLURP-2 ≥ rSLURP-1 (Fig 4C).

## Discussion

The progress in molecular biology and studies of ligand-receptor interactions in particular is tightly coupled with the ability to produce recombinant analogues of the proteins which otherwise could not be obtained from natural sources in sufficient amounts [29,30]. Frequently, the recombinant proteins differ from the native ones and contain cloning artifacts, stabilizing mutations, purification tags, or non-native glycosylation patterns. These ‘modifications’ could affect the functional and structural properties of a studied protein.

It has been demonstrated that the hybrid protein SUMO-SLURP-1 (MW ~ 22 kDa) inhibited proliferation of oral keratinocytes (Het-1A cells) [12], and that a recombinant SLURP-1 analogue containing an unprocessed *N*-terminal signal peptide (103 a.a., MW ~ 11 kDa, [22]) induced upregulation of nuclear factor-κB gene expression in keratinocytes [13]. Moreover, the transfection of Het-1A cells with siRNA-α7 or inhibition of the α7-nAChRs with selective antagonist methyllycaconitine has resulted in the disappearance of the effects induced by this SLURP-1 analogue [13]. As a result of above mentioned studies Grando et al have proposed that the effects of native SLURP-1 on keratinocytes could be mediated by agonistic interaction with α7-nAChRs [12,13].

In agreement with these findings, we also observed inhibition of keratinocyte proliferation by rSLURP-1 (Fig 1, 82 a.a., MW 8,974 Da). We further demonstrated that the effect of rSLURP-1 was concentration-dependent with pronounced antiproliferative activity at nanomolar concentrations (EC_50_ of ~4 nM, Fig 1B). The incubation of keratinocytes with anti-α7-nAChRs antibodies significantly diminished the rSLURP-1 activity (Fig 1C). However, the preincubation of the cells with α-Bgtx (selective antagonist of α7-nAChRs) did not influence the rSLURP-1 antiproliferative effect (Fig 1C). This discrepancy forced us to investigate in details the SLURP-1 interaction with its potential molecular targets.

There are two classes of acetylcholine receptors participating in cholinergic signaling: nicotinic receptors, which are ligand-gated ion channels, and muscarinic receptors, which are G-protein coupled receptors (GPCRs). Keratinocytes express both types of acetylcholine receptors [28]. Some structural homologues of SLURP-1, e.g. non-conventional ‘three-finger’ snake toxin WTX [31] and ws-Lynx1 [26] (Fig 1A), are able to interact with both classes of receptors, nAChRs and mAChRs. Therefore we decided to study whether SLURP-1 displays similar functional dualism. Analysis of the rSLURP-1 antiproliferative effect in the presence of Mec and atropine,—non-selective inhibitors of nAChRs and mAChRs, respectively, revealed nicotinic receptors as a rSLURP-1 target in keratinocytes (Fig 1C).

To further characterize the potential targets of rSLURP-1 we performed affinity purification of different nAChR subunits using the bead-coupled ligand. Due to a low expression of nicotinic receptors on keratinocytes [28] we used a different tissue: cortical extracts from the human brain. Using this approach we demonstrated for the first time that rSLURP-1 exclusively binds to α7 nAChR subunit (Fig 2). This is strikingly different from the selectivity pattern of ws-Lynx1. In similar experiments, ws-Lynx1 demonstrated the interaction with α3, α4, α5, α6, α7, β2 and β4 nAChR subunits [32]. Thus, our results highlight an exclusive targeting by SLURP-1 of α7 nAChRs.

Electrophysiology experiments revealed that rSLURP-1 diminishes the current amplitudes of α7-nAChRs expressed in *Xenopus* oocytes, with quite low efficiency (IC_50_ of ~1 μM). Notably, the inhibitory effect of rSLURP-1 is increased with increasing ACh concentrations (Fig 3). Enhancement of rSLURP-1 effect in the presence of high agonist (ACh) concentrations is consistent with open channel block (the more receptors are activated by agonist, the more of them are inhibited by rSLURP-1). At the same time the direct blockage of the α7-nAChR channel by large ‘three-finger’ molecule seems to be sterically impossible. The similar ‘blocker-like’ behavior is not unusual for the nAChR ligands. The well-known nAChR inhibitor d-tubocurarine also has activity of open-channel blocker [33], nevertheless only intersubunit binding sites at the extracellular part of the receptor have been identified so-far [34]. The non-complete (up to ~ 70%) inhibition of the ACh-evoked currents observed at the high concentrations of rSLURP-1 argues in favor of allosteric mode of interactions with the receptor. We therefore hypothesize that SLURP-1 acts as an allosteric modulator preventing hyperactivation of Ca^2+^-conducting α7-nAChRs thus protecting Ca^2+^-dependent signaling pathways from undesirable activation.

It has been previously reported that a SLURP-1 variant with a *C*-terminal Myc-tag acted on α7-nAChRs at nanomolar concentrations by increasing their current amplitudes [10]. Here we did not observe an enhancement of current amplitudes by application of rSLURP-1 over a wide range of concentrations (Fig 3B). This discrepancy could be explained by the sequence difference of the SLURP-1 variants. The Myc-tag consists of 10 residues (EQKLISEEDL), and five of them are charged. The involvement of long *C*-terminal tail of the ‘three-finger’ snake α-neurotoxins in the interaction with α7-nAChRs has been previously described [35,36]. It is therefore possible that this relatively long negatively charged *C*-terminal sequence influences the properties of SLURP-1 and the mode of interaction with the receptor.

We observed competition between rSLURP-1 and α-Bgtx for binding to muscle type nAChR and AChBP (Fig 4A, [23]). These data suggest that the rSLURP-1 binding site at least partially overlaps with the α-neurotoxin binding sites on these targets. Contrary to that, no rSLURP-1 competition with α-Bgtx for binding with α7-nAChRs was detected in GH_4_C_1_ cells overexpressing these receptors (Fig 4B). At the same time, the affinity purification revealed that rSLURP-1 binds to α7-nAChR subunit in these cells although with lower affinity than α-Bgtx (Fig 4C). The affinity of rSLURP-1 to α7-nAChR subunit was comparable or slightly less than affinity of rSLURP-2. Taking in to account that rSLURP-2 acts on α7-nAChRs at nanomolar concentrations (Lyukmanova et al, unpublished data) we could not exclude that the rSLURP-1 also has nanomolar affinity to α7-nAChRs.

The absence of competition between rSLURP-1 and ACh was revealed by electrophysiology on *Xenopus* oocytes expressing α7-nAChRs (Fig 3D). Taken together with data obtained on α7-nAChRs overexpressed in GH_4_C_1_ cells, these findings demonstrate that rSLURP-1 binds to α7-nAChRs outside the classical agonist/antagonist binding site. Similar results have been recently reported for ws-Lynx1, which at concentration of 10 μM inhibited currents through α7, α4β2, and α3β2 receptors, but did not compete with ^125^I-α-Bgtx and ^3^H-epibatidine for binding to α7- and α4β2-nAChRs at concentrations up to 30 μM [26].

Several effects could be responsible for the observed two-order of magnitude difference in the effective rSLURP-1 concentrations in various assays (~ 4 nM for inhibition of keratinocytes proliferation and ~1 μM for suppression of ionic currents at α7-nAChRs). First of all, we cannot exclude the possibility that α7 receptors of keratinocytes have different properties from the receptors expressed in *Xenopus* oocytes. For instance, the observed difference could arise from complex interactions of rSLURP-1 with various states of α7-nAChR in keratinocytes (coupled, uncoupled, desensitized etc.). However, the provocative idea that the inhibition of keratinocyte proliferation by rSLURP-1 is not connected with the modulation of ionic currents through the Ca^2+^-conducting α7-nAChRs seems also possible. This hypothesis is supported by the absence of antiproliferative activity of the nAChR inhibitors (Mec and α-Bgtx, Fig 1C) and of ws-Lynx1, which demonstrates modulatory activity on α7-nAChR currents that is comparable to that of rSLURP-1 (Figs 1B and 3C). We could hypothesize that rSLURP-1 activity in keratinocytes is related to ‘non-classical’ signaling pathway through α7-nAChR, that activates intracellular signaling cascades without opening the receptor channel (metabotropic type of signaling). It is possible that SLURP-1 has two different binding sites on α7-nAChR, and that binding to the ‘high-affinity’ site mediates metabotropic type of signaling, whereas biding to the ‘low-affinity’ site is responsible for the regulation of ion flow through the receptor channel. This would explain the responses of keratinocytes to co-application of SLURP-1 with different α7-nAChR inhibitors: the non-specific blocker of nAChR Mec inhibits both metabotropic and ionotropic pathways, while competitive antagonist α-Bgtx blocks only ionotropic events and thus does not influence rSLURP-1 antiproliferative activity. The inhibition of the rSLURP-1 antiproliferative effect by anti-α7-nAChR antibodies could be explained by sterical hindrances induced by the large antibody molecule, which could shield the rSLURP-1 binding sites on the receptor surface.

A possible effect of SLURP-1 on metabotropic signaling through the α7-nAChR has recently been suggested by Grando et al, who observed that the inhibition of Ca^2+^ passage through α7-nAChRs in Ca^2+^-free medium or by Cd^2+^ or Zn^2+^ ions only partially suppress the SLURP-1 effects in keratinocytes [13]. According to recent reports, metabotropic signaling through nAChRs could involve different effector molecules: Jak2 kinase [13], EGF and VEGEF receptor tyrosine kinases [37], and G-proteins [38]. Probably these molecules could sense the changes in the cytoplasmic part of the nAChRs arisen from SLURP-1 binding to extracellular part of the receptor, and transmit the signal further to other cytoplasmic targets.

## Conclusions

Engineering of the bacterial expression system for production of rSLURP-1 with near-native structure (the only difference is the addition of *N*-terminal Met) [23] allowed us to obtain new information about the function of this protein. For the first time we demonstrated that rSLURP-1 binds selectively to the α7 subtype of nicotinic acetylcholine receptors. rSLURP-1 does not compete with α-bungarotoxin for binding to GH_4_C_l_ cells expressing α7-nAChRs, but inhibits α7-nAChRs expressed in *Xenopus* oocytes acting as allosteric antagonist. rSLURP-1 reduces proliferation of human keratinocytes, and the antiproliferative activity of rSLURP-1 is mediated by interaction with α7-nAChRs, but not suppressed by α-bungarotoxin. Our data support the hypothesis that the effects of SLURP-1 in the cells are mediated by a ‘non-classical’ mode of interaction with α7-nAChR, which may rely on metabotropic signaling. We hope that future structural-functional studies will give an opportunity to elucidate possible molecular partners involved in this type of nAChR signaling and shed light on the detailed molecular mechanisms of SLURP-1/receptor interaction.

## Abbreviations

ACh: acetylcholine
α-Bgtx: α-bungarotoxin
Ls-AChBP: *Lymnaea stagnalis* acetylcholine binding protein
mAChR: muscarinic acetylcholine receptor
Mec: mecamylamine
nAChR: nicotinic acetylcholine receptor
rSLURP-1: recombinant analogue of human SLURP-1
ws-Lynx1: water-soluble domain of human Lynx1
